# Long non‐coding RNAs in brain tumours: Focus on recent epigenetic findings in glioma

**DOI:** 10.1111/jcmm.13781

**Published:** 2018-08-17

**Authors:** Sevinci Pop, Ana‐Maria Enciu, Laura G. Necula, Cristiana Tanase

**Affiliations:** ^1^ “Victor Babes” National Institute of Pathology Bucharest Romania; ^2^ “Carol Davila” University of Medicine and Pharmacy Bucharest Romania; ^3^ “Stefan N. Nicolau” National Institute of Virology Bucharest Romania; ^4^ Faculty of Medicine “Titu Maiorescu” University Bucharest Romania

**Keywords:** cancer stem cells, CRNDE, epigenetic, glioblastoma, glioma, long non‐coding RNAs, TUNA

## Abstract

Glioma biology is a major focus in tumour research, primarily due to the aggressiveness and high mortality rate of its most aggressive form, glioblastoma. Progress in understanding the molecular mechanisms behind poor prognosis of glioblastoma, regardless of treatment approaches, has changed the classification of brain tumours after nearly 100 years of relying on anatomopathological criteria. Expanding knowledge in genetic, epigenetic and translational medicine is also beginning to contribute to further elucidating molecular dysregulation in glioma. Long non‐coding RNAs (lncRNAs) and their main representatives, large intergenic non‐coding RNAs (lincRNAs), have recently been under scrutiny in glioma research, revealing novel mechanisms of pathogenesis and reinforcing others. Among those confirmed was the reactivation of events significant for foetal brain development and neuronal commitment. Novel mechanisms of tumour suppression and activation of stem‐like behaviour in tumour cells have also been examined. Interestingly, these processes involve lncRNAs that are present both during normal brain development and in brain malignancies and their reactivation might be explained by epigenetic mechanisms, which we discuss in detail in the present review. In addition, the review discusses the lncRNAs‐induced changes, as well as epigenetic changes that are consequential for tumour formation, affecting, in turn, the expression of various types of lncRNAs.

## 1 INTRODUCTION

Twenty‐first‐century medical diagnostics are shifting from clinical and anatomical pathology towards molecular‐genetic, epigenetic, transcriptomic and proteomic methodologies. Detection of molecular alterations is currently included as diagnostic criteria in various areas of clinical oncology, such as breast cancer^1^ and myeloid neoplasms,^2^ and “clinical sequencing” is being implemented in oncology practices worldwide.^3^ Beyond genetic mutations, epigenetic alterations, such as DNA methylation and modification of histone codes, or dysregulated expression of non‐coding RNAs, could affect the regulation of gene expression, further contributing to tumour heterogeneity.^4^, ^5^

Gliomas are the most common type of brain malignancies and are accompanied by difficult diagnosis due to their high inter‐ and intratumoral heterogeneity. Glioblastoma multiforme (GBM) is the most heterogeneous and incurable glioma subtype. Recently, the WHO classification has been modified to include molecular criteria, such as IDH mutations for diffuse astrocytoma and GBM, or the epigenetic mark H3K27M mutation for diffuse midline glioma, to provide more precise diagnosis and treatment.^6^ However, the quest for potential therapeutic targets remains unfulfilled;^7^, ^8^ thus, new strategies are emerging to explore the molecular basis of brain tumour development for clinical exploitation.

An expanding research area in epigenetic regulation of tumorigenesis includes the field of non‐coding RNAs. Among non‐coding species, lncRNAs are being intensively investigated, and enticing progress has been made in recent years, revealing their roles in chromatin remodelling, transcription, posttranscriptional processing and intracellular trafficking.^9^, ^10^ lincRNAs consist of separate transcript units that are located between, but do not overlap with, protein‐coding genes (PCGs) and represent the largest subclass of functional characterized lncRNAs.^11^ lincRNAs have highly tissue‐specific expression and are primarily involved in epigenetic regulation of PCG expression as well as in normal development processes, including embryogenesis, stem cell pluripotency and differentiation.^12^, ^13^

Aberrant expression of lncRNAs is associated with several types of cancer, including glioma. Normal expression of lncRNAs is affected by functional mutations or epigenetic alterations, transforming them into cancer‐associated transcripts present at every step of tumour development. Several lincRNAs have been associated with oncogenic mechanisms in gliomas, such as interference with glial cell differentiation and maintenance of stemness in glial cancer stem cells, detailed later in the present review. The differential expression patterns of lncRNAs between tumour and normal tissues, along with expression discrepancies in tumours with different clinical features, suggest that lncRNAs could act as diagnostic and prognostic biomarkers and pharmaceutical targets in gliomas.

This review focuses on lncRNA‐related mechanisms found to be activated in glioma molecular biology, some of which are significant to the developmental biology of the central nervous system, becoming aberrantly reactivated in adulthood. Several lncRNAs involved in embryogenesis that are found to be reactivated in brain tumour are discussed in the section dedicated to lncRNA functions in glioma biology. We will also review the two sides of lncRNA epigenetic regulation in glioma, as both targets and drivers. Finally, arguments in favour of using lncRNAs as diagnostic tools and therapeutic targets in glioma will be presented.

## 2 BASIC CHARACTERISTICS AND FUNCTIONS OF LNCRNAS

Completion of human genome sequencing and de novo mammalian transcriptome characterization has revealed tens of thousands of lncRNA sequences emanating from uncharacterized genomic regions.^14^, ^15^ LncRNA transcripts are lengthy (>200 nucleotides), often containing multiple functional domains capable of interacting with DNA, proteins or RNAs (mRNAs and microRNAs). The versatility of lncRNAs to fold into a variety of secondary and tertiary structures explains the diversity of their interacting partners and the complexity of their molecular functions.

Four types of molecular mechanism for lncRNAs have been distinguished, whereby lncRNAs act as signals, decoys, guides or scaffolds.^16^, ^17^ Signal lncRNAs regulate transcriptional activity or signalling initiation. Decoy lncRNAs bind and titrate away gene regulatory elements (proteins, mRNAs, microRNAs). In the nucleus, lncRNAs can bind transcription factors (TFs) or chromatin modifiers, whereas in the cytoplasm, they function as a sponge to attract proteins and miRNA/RISC complexes away from their targets. In Table 1, we summarized some of significant lncRNA‐miRNA interactions with impact on glioma biology and pathology, where lncRNA is acting as competing endogenous to sponge miRNAs. Their interactions are discussed in detail in the next sections. Guide lncRNAs direct their molecular target (proteins) either in *cis* on neighbouring genes or in *trans* to distantly located genes. Scaffold lncRNAs bind and assemble multiple effector partners (proteins, RNAs) into complexes, controlling their formation and localization.^18^, ^19^

**Table 1:** lncRNA‐miRNA interactions with role in glioma pathology and biology

lncRNA expression	Type of lncRNA‐miRNA interaction	miRNA expression	Function of miRNA in glioma biology	Changes in miRNA levels with glioma grading	References
CRNDE ↑	ceRNA‐molecular sponge to miRNAs	miR‐136‐5p **↓** miR‐186 **↓** miR‐384 **↓**	Tumour suppressors	**↓** Glioma grade I‐IV	31, 51, 52, 100, 101
Linc NEAT1 ↑	ceRNA‐molecular sponge to miRNAs	miR‐181d‐5p **↓** miR‐449b‐5p **↓**	Modulates the MGMT expression at protein level and mRNA Modulates c‐Met expression	**↓** GBM in comparison with normal brain samples	44, 57, 58
SOX2OT ↑	ceRNA‐molecular sponge to miRNAs	miR‐122 **↓** miR‐194‐5p ↓	Tumour suppressors	**↓** Glioma grade I‐IV	53, 54
Linc H19 ↑	ceRNA‐molecular sponge to miRNA ceRNA‐molecular sponge to miRNA H19 is a precursor of miR‐675	let‐7 **↓** miR‐29a **↓** miR‐675 **↑**	Tumour suppressor Tumour suppressor Oncogene	**↓** Glioma grade I‐IV **↓** Glioma grade I‐IV **↑** Glioma grade I‐IV	46, 62, 102, 103
TUG1 ↑	ceRNA‐molecular sponge to miRNAs	miR‐145 **↓** miR‐299 **↓**	Tumour suppressors	**↓** Glioma grade I‐IV **↓** GBM in comparison with normal brain samples	46, 64, 67
Linc XIST ↑	ceRNA‐molecular sponge to miRNAs	miR‐137 **↓** miR 152 **↓**	Tumour suppressors	**↓** Glioma grade I‐IV	22, 46, 71
Linc‐ROR ↓	ceRNA‐molecular sponge to miRNA	miR‐145 **↑**	Tumour suppressor	**↓** Glioma grade I‐IV	77, 78, 79

ceRNA, competing endogenous RNA; **↑,** increased expression of RNA species; **↓,** decreased expression of RNA species; GBM, glioblastoma multiforme (WHO grade IV glioma); MGMT, O‐6‐methylguanine‐DNA methyltransferase; c‐Met, MET proto‐oncogene, receptor tyrosine kinase.

Based on these characterized mechanisms, lncRNAs can initiate regulatory networks with high complexity at epigenetic, transcriptional and posttranscriptional levels required for cellular functions. Though lncRNAs exhibit poor sequence conservation and are less expressed than are PCGs, they present the highest specificity with respect to cell type, subcellular compartment, developmental stage and in response to environmental stimuli.^11^, ^20^, ^21^

LncRNAs play regulatory and structural roles in diverse cellular processes, including embryogenesis, stem cell pluripotency, differentiation and senescence. Interestingly, compared to that in other organs, the highest number of expressed lncRNAs has been found in the brain, and their number surpasses that of the brain PCG transcripts.^22^, ^23^ The brain's lncRNAs have the highest tissue specificity and are the most evolutionarily conserved, with similar spatiotemporal expression patterns across multiple species.^23^, ^24^, ^25^

In neural development, lncRNAs have important roles in regulating stem cell maintenance and differentiation programmes, including cell fate specification^26^ and neural lineage commitment.^27^, ^28^ Under a complex programme of differentiation, partially controlled by lncRNAs, diverse types of neuroprogenitors develop into different neuronal and glial cell subtypes.^29^ Moreover, differentially expressed lncRNAs across various stages of differentiation indicate that they can amplify and consolidate the molecular differences between cell types that are required to control cell identity and lineage commitment.^30^

Some of these embryonically active programmes are reactivated during adulthood, primarily during oncogenic transformation, which will be discussed in this review.

## 3 DYSREGULATED LNCRNA EXPRESSION IN GLIOMA PATHOLOGY

Systemic high‐throughput studies, including lncRNA microarray and RNA sequencing on hundreds of classified glioma samples and normal brain tissues, have demonstrated that lncRNAs, in addition to their involvement in normal biological processes, represent key players during tumorigenesis.^31^

Zhang et al^32^ identified 129 lncRNAs differentially expressed between glioma and normal brain tissues. Two lncRNAs, colorectal neoplasia differentially expressed (CRNDE) and HOX antisense intergenic RNA myeloid 1 (HOTAIRM1), showed the highest expression in glioma (grade I‐IV) compared with that in normal tissue. Interestingly, both lncRNAs are also involved in brain development and neuronal differentiation,^31^, ^33^ with HOTAIRM1 highly expressed in foetal brain.^34^ Comparing recurrent glioma samples with primary tumours, CRNDE and HOTAIRM1 were significantly up‐regulated among thousands of differentially expressed lncRNAs. The same study demonstrated that many dysregulated lncRNA‐mRNA pairs from the recurrent group were closely related to cancer or neural differentiation.^35^

Several studies have correlated lncRNA expression profiles with different histological subtypes and malignancy grades in gliomas.^32^, ^36^, ^37^ Global gene expression analyses identified 27 lncRNAs that are differentially expressed between astrocytomas and oligodendrogliomas.^32^ For GBM, six lncRNAs were found to be significantly associated with patient prognosis but were independent of patient age or MGMT promoter methylation status.^38^ One transcript, KIAA0495, was abundantly expressed in GBM tissues and associated with reduced survival. Myocardial infarction‐associated transcript (MIAT/Gomafu), a neuron‐specific component of the nuclear matrix^39^ involved in neurogenesis and neural stem cells differentiation,^40^ was identified as a significantly down‐regulated lncRNA in glioma.

Gliomas can be classified into three different groups based on their dysregulated pattern of lncRNA expression and may be further associated with mutational status, molecular subtypes and clinical outcome.^41^ In addition, an individual lncRNA can indicate stages in tumour progression and might be useful as an independent biomarker for diagnosis and prognosis. For example, the HOX transcript antisense RNA (HOTAIR) exhibits reduced expression in low‐grade gliomas (LGG) compared with that in GBM. Furthermore, differentiated levels of expression between GBM subtypes were revealed, with higher levels of HOTAIR in classical and mesenchymal subtypes than those in proneural, a neural subtype.^37^

Recently, hundreds of lncRNAs differentially expressed between glioma samples (grades I‐IV) and normal brain tissues were identified and were subsequently associated with glioma pathology.^42^ Among them, CRNDE was found to exhibit a 40‐fold higher expression in GBM than that in normal tissues. Additionally, TUNA (TCL1 upstream neural differentiation‐associated RNA) was shown to be severely down‐regulated in all glial tumours by 45‐fold in GBMs and 14‐fold in LGGs. TUNA performs a regulatory function in pluripotency and neural differentiation of ESCs, acting as a scaffold for RNA‐binding proteins. TUNA also regulates the expression of several key neurogenic genes, including SOX2, and its depletion causes down‐regulation of SOX2 and subsequent loss of neurogenesis.^43^ Therefore, TUNA represents another example of an lncRNA involved in both neurogenesis and brain tumour progression.

Using a nONCOchip custom array for astrocytoma, Hackermuller et al demonstrated that approximatively 40% of the total identified transcripts exhibited different expression levels between astrocytoma grades. Interestingly, a part of those transcripts was expressed in response to cell cycle, p53 and STAT3 pathway activation.^44^ A novel four‐lncRNA signature that accurately predicts survival in GBM patients was recently reported, and results of a functional analysis suggest that co‐expressed genes tend to cluster within nine immune‐related processes and four biological pathways.^45^

**Figure 1:**
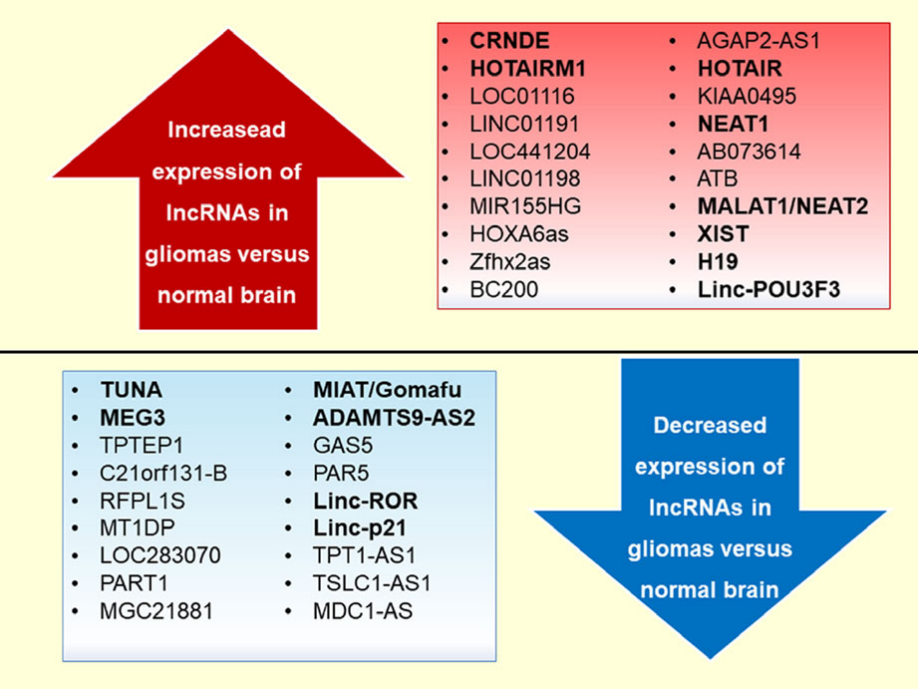
Graphical Representation of Differential Expressed lncRNAs in Glioma Tissues in Comparison with Normal Brain Samples; the Bolded LncRNAs are also Present in Normal Brain Development

While dysregulation of lncRNAs has been correlated with glioma pathology and alteration of diverse signalling pathways, their functional significance in cancer is only beginning to be explored. Importantly, identification of key lncRNAs involved in neurogenesis and normal brain development (e.g CRNDE, HORAIRM1 and TUNA) as the most dysregulated lncRNAs in glioma suggests aberrant reactivation as a mechanism that promotes oncogenesis (Figure 1).

## 4 FUNCTIONS OF LNCRNAS IN GLIOMA BIOLOGY

The hallmark of brain tumours includes rapid cell proliferation, resistance to apoptosis, invasion of surrounding brain tissue, elevated levels of angiogenesis and the existence of therapy‐resistant GSCs. The functional role of lncRNAs is beginning to be validated by systematic experimental analysis of lncRNA activity and effects in normal and transformed cells, including both in vitro and in vivo models.

Table 2 shows functionally validated lncRNAs involved in embryogenesis that have also been identified as oncogenic or tumour suppressors in glioma cancer biology.^31^, ^46^, ^47^, ^48^, ^49^

**Table 2:** LncRNAs involved in embryogenesis and in glioma biology

LncRNA	Changes	Reported function in embryogenesis	Reported function in glioma biology	Molecular mechanism	Potential application	References
Linc CRNDE	Up‐regulated in glioma (grades I‐IV)	Neurogenesis, neuronal and iPSC differentiation	Promotes glioma cell growth and migration Regulation of GCSs	Epigenetic dysregulation in glioma Scaffold with PRC2 and CoREST	Potential biomarker, associated with glioma grading Potential therapeutic target	31, 32, 33, 35, 37, 51, 52
Linc MIAT (Gomafu)	Down‐regulated in glioma	Neurogenic commitment Neuronal survival	Correlated with neurovascular dysfunction	Interacts mainly with RNA binding proteins	Associated with prolonged survival	38, 40, 77
HOTAIRM1	Up‐regulated in glioma	Neurogenesis and brain development Neuronal differentiation	Potential roles in glioma‐genesis and development	Scaffold with PRC1 and PRC2 Epigenetic dysregulated in GBM Marked by H3K4me3 in GSCs	Potential biomarker	30, 31, 32, 34, 35
HOTAIR	Up‐regulated in GBM Down‐regulated in LGG	Development and differentiation	Promotes glioma cell growth, GSC maintenance Promotes cellular processes: Cell cycle progression, migration, invasion, metastasis	Regulated by BRD4 protein Scaffold with PRC2 and LSD1/CoREST/REST complex	Biomarker for glioma prognosis, overall survival rate and for GBM molecular subtype classification Potential therapeutic target	37, 93, 94
Linc NEAT1	Up‐regulated in solid tumours, including glioma	Essential in nuclear paraspeckle formation Role in neuronal and glial cell differentiation	Promotes glioma cell proliferation, invasion and migration Promotes GSC malignancy Modulates BTB permeability	Scaffolding PRC2 complex (EZH2)	Biomarkers for GBM prognosis and therapy resistance Potential therapeutic target	40, 57, 58, 60
Linc MALAT1/NEAT2	Upregulated in GBM and GSCs	Role in neuronal and glial cell differentiation	Brain cancer initiation and progression Promotes GSC invasion	Regulated by WIF1 expression via the WNT5A/p38‐MAPK/Ca2 + ‐non‐canonical WNT signaling axis	Prognostic factor Potential therapeutic target	40, 45, 59, 60
SOX2OT	Up‐regulated in glioma and GSCs	Neurogenesis	Promotes proliferation, migration and invasion of GSCs Inhibits apoptosis of GSCs	Interacts with TFs involved in pluripotency Interaction with PRC2 complex (EHZ2) Interacts with cancer associated genes ROCK2 and NFKB	Potential therapeutic target	34, 54
Linc H19	Up‐regulated in GBM tissues and GCSs	Role in genomic imprinting during foetal growth, Role in maintenance of the stemness in embryonic stem cells Prevents and regulates myoblast differentiation	Promotes glioma progression Promotes invasion, angiogenesis, stemness and tumorigenicity of GBM and inhibits apoptosis of GBM cell lines Maintains hypoxia in GBM	Binds to c‐Myc Sponges the pro‐differentiation let7 miRNA family Binds to Hif‐1α	Potential therapeutic target	46, 50, 61, 62
TUG1	Up‐regulated in glioma, GBM and GSCs	Epigenetic regulator of neuronal differentiation‐associated genes Development of retinal cells	Promotes proliferation and invasion of glioma cells and inhibits apoptosis Enhances VEGF expression and angiogenesis in GBM Induces GSC differentiation and self‐renewal	Interacts with SOX2, c‐MYC Scaffold for PRC2 complex, guide for EZH2	Therapeutic target for enhancing blood‐tumour barrier permeability	46, 64, 67
Linc XIST	Up‐regulated in GBM tissues and GSCs	X chromosome inactivation	Promotes GSCs proliferation, migration, invasion Promotes glioma angiogenesis and BTB	Scaffold for PRC2 complex Guides the deposition of H3K27me3 epigenetic mark to silence transcription	Potential therapeutic target	22, 46, 71
Linc‐POU3F3	Up‐regulated in glioma tissues and GSCs	Regulation of key genes in the neurogenic differentiation of stem cells; required to maintain the undifferentiated phenotype of neural precursors	Promotes glioma cell proliferation	Interacts with EZH2, epigenetically modulates neighboring POU3F3 gene expression	Potential therapeutic target	72, 73
Linc TUNA	Down‐regulated in all glial tumours	Functions in pluripotency and neural differentiation Regulates neural gene promoters	Possible regulator for oncogenes in gliomas	Binds to SOX2 and NANOG Scaffold for RNA‐binding proteins	Potential therapeutic target	38, 43
Linc‐ROR	Down‐regulated in glioma and GSCs	Controls ESC self‐renewal and promotes cells reprogramming Involved in maintaining pluripotency Negative regulator of p53	Inhibits glioma cell proliferation Inhibits proliferation, self‐renewal and spheronization of GSCs	Interacts directly with iPSC TFs (SOX2, OCT4, NANOG, KLF4) Interacts with EZH2	Potential therapeutic target	77, 78, 79
Linc MEG3	Down‐regulated in glioma tissues and GBM	Maternally imprinted gene, present in adult brain	Inhibits proliferation of glioma cells and induces apoptosis Involved in angiogenesis and brain vascularization	Regulated by DNA methylation and DNMT1 Binds to p53 protein	Potential therapeutic target Biomarker for GBM prognosis	32, 47, 61, 74
ADAMTS9‐AS2	Down‐regulated in glioma	Antisense transcript of ADAMTS9 gene that is widely expressed in foetal and adult tissues	Inhibits migration and invasion of glioma cells Inhibits GSC malignancy	Regulated by DNA methylation and DNMT1	Marker for tumour grade and prognosis Potential therapeutic target	75, 76
Linc‐p21	Down‐regulated in glioma and GSCs	Prevents iPSCs reprogramming Represses p53‐interfering genes to regulate cellular apoptosis	Inhibits GCSs proliferation, self‐renewal and glycolysis and anti‐EMT activity	A p53‐dependent transcriptional target gene Scaffold with SETDB1(H3K9 methyltransferase) and DNMT1	Potential therapeutic target	30, 80, 81

Linc CRNDE, colorectal neoplasia differentially expressed; Linc MIAT (Gomafu), myocardial infarction‐associated transcript; HOTAIRM1, HOX antisense intergenic RNA myeloid 1; HOTAIR, HOX antisense intergenic RNA; Linc NEAT1, nuclear paraspeckle assembly transcript 1; Linc MALAT1/NEAT2, metastasis‐associated lung adenocarcinoma transcript 1/nuclear paraspeckle assembly transcript 2; SOX2OT, SOX2 overlapping transcript; TUG1, taurine up‐regulated 1; Linc XIST, X‐specific inactive transcript; Linc‐POU3F3, POU Class 3 homeobox 3 intergenic RNA; Linc TUNA, Tcl1 upstream neuron‐associated/MEGAMIND; Linc‐ROR, regulator of reprogramming; Linc MEG3, maternally expressed 3 long intergenic RNA; ADAMTS9‐AS2, ADAMTS9 antisense RNA 2.

## 5 ONCOGENIC lncRNAS

CRNDE, one of the most highly expressed lncRNAs during neuronal differentiation, in induced pluripotent stem cells (iPSCs) and within gliomas^32^, ^35^, ^50^ exerts its oncogenic function by promoting glioma cell growth, invasion and migration through different signalling pathways.^51^ Acting as a decoy, lncRNAs support glioma progression by reducing miR‐136‐5p expression, directly impacting PI3K/AKT/mTOR signalling pathways.^52^ Knockdown of CRNDE combined with overexpression of miR‐384 restrained glioma tumour growth and increased survival in a nude mouse model. CRNDE promotes GSC malignancy by negatively regulating miR‐186.^31^ Interestingly, the expression of CRNDE in stem cells is regulated by c‐Myc, a multipotent TF that stimulates gene amplification and is overexpressed in many cancers, including glioma.^50^

SOX2OT is involved in neurogenesis and glioma development.^53^ Silencing SOX2OT inhibits proliferation, migration and invasion of GSCs and promotes their apoptosis. A direct interaction of SOX2OT with two tumour‐suppressing miRNAs and their subsequent negative regulation was demonstrated. Su et al proved the existence of SOX2OT‐miR‐194‐5p/miR‐122‐SOX3‐TDGF‐1 pathway that forms a positive feedback loop that regulates the biological behaviours of GSCs.^54^ Importantly, SOX2OT is highly evolutionarily conserved and interacts with master regulators of pluripotency and with cancer‐associated genes such as ROCK2 and NFKB. Patterns of concomitant expression of SOX2OT and SOX2 in stem cells and several human cancers suggest a common co‐regulation mechanism and involvement of similar molecular pathways.^55^

**Figure 2:**
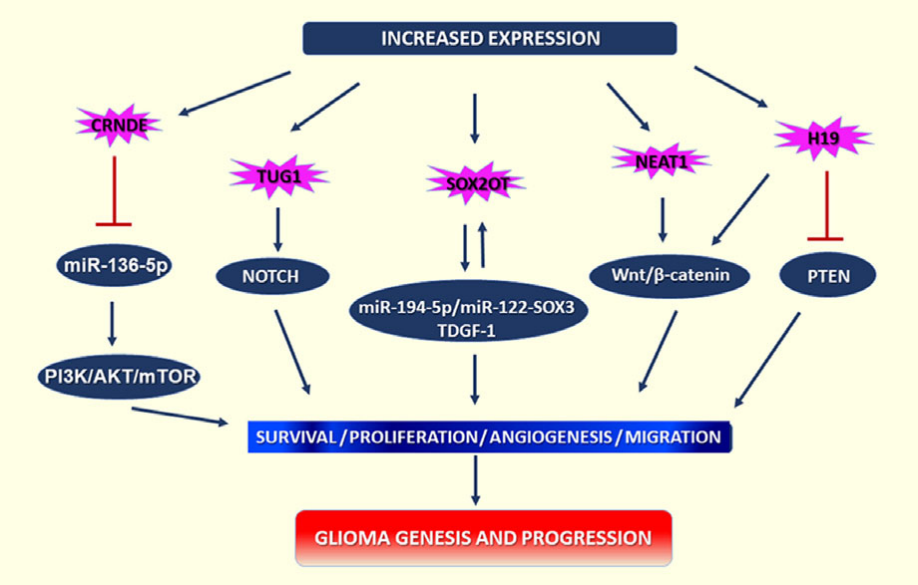
Mechanism of lncRNAs Involvement in Signalling Pathways

NEAT1 is essential for nuclear paraspeckle formation and is overexpressed in most solid tumours.^56^ The expression of NEAT1 is closely correlated with higher WHO grade and recurrence in gliomas.^31^ Oncogenic NEAT1 depletion through the dual‐CRISPR/Cas9 system inhibited GBM cell growth and invasion both in vitro and in vivo. NEAT1 expression is regulated by EGFR pathway activity. Additionally, NEAT1 silencing in GBM cells affects the Wnt/β‐catenin pathway, which is associated with early embryonic patterning and regulation of stem cell self‐renewal and differentiation.^57^ NEAT1 promotes malignant progression of GSCs by sponging miRNAs and modulates the permeability of BTB by binding miR‐181d‐5p and affecting its downstream targets, ZO‐1, occludin and claudin‐5.^58^ Nuclear MALAT1/NEAT2 is one of the most highly conserved lncRNAs among mammals and is significantly up‐regulated in primary tissues and serum samples from GBM patients who exhibit resistance to TMZ‐based treatment.^45^ Furthermore, MALAT1 is more highly expressed in GSCs than it is in glioma tissues in general. Oncogenic MALAT1 exerts a strong positive effect on GSC invasion, and its expression is regulated by WIF1 expression via the WNT5A/p38‐MAPK/Ca2 + ‐non‐canonical WNT signalling axis^59^ (Figure 2).

Both nuclear transcripts NEAT1 and NEAT2 have essential roles in normal development for muscle, neuronal and glial cells differentiation^40^, ^60^ and might be involved in glioma iniation and progression as well.

LincRNA H19 is abundantly maternally expressed in embryonic tissues and plays a significant role in GBM development. H19 has multiple validated oncogenic functions, such as promoting invasion, angiogenesis, stemness and tumorigenicity of GBM cells. Its expression in GBM is induced by proto‐oncogene c‐MYC, and H19 can further modulate glioma progression by generating the oncomiR‐675, which has as its direct target the cancer‐associated cadherin 13 (CDH13), a tumour‐suppressor protein in several types of cancer.^46^ H19 knockdown in GBM cell lines was shown to increase TMZ‐induced apoptosis and down‐regulation of GSCs markers, such as CD133, NANOG, OCT4 and SOX2.^61^ Recently, hypoxia‐induced H19 expression has been reported. The binding of hypoxia‐inducible factor 1α (Hif‐1α) to the H19 promoter induces a migratory and invasive phenotype in GBM cells. Interestingly, some EMT‐related proteins, including β‐catenin and PTEN, are also regulated by H19 during hypoxia.^62^, ^63^

TUG1 is expressed during retinal development and has numerous biological functions in cancer, acting primarily as an oncogene. Overexpression of TUG1 in glioma cells leads to the alteration of apoptosis‐related mediator proteins.^46^ In addition, TUG1 is a downstream effector of the p53‑regulatory network, promoting proliferation and invasion of glioma cells and inhibiting apoptosis. In a xenograft GBM model, TUG1 was shown to enhance tumour‐induced angiogenesis and VEGF expression through inhibition of miR‐299.^64^ More importantly, TUG1 is an inducer of GSC differentiation via the Notch pathway, whose increased activity promotes brain tumour growth.^65^, ^66^ TUG1 coordinately promotes self‐renewal by sponging miR‐145, a tumour suppressor miRNA, and regulates the stemness‐associated genes SOX2 and c‐Myc. Furthermore, intravenous treatment with antisense oligonucleotides targeting TUG1 induces GSCs differentiation and efficiently represses GSC growth in vivo.^67^

LOC441204 is another lncRNA overexpressed in brain tumour and glioma cell lines, and the pro‐oncogenic role of LOC441204 in tumour cell growth appears to be associated with the activation of the β‐catenin/p21/cdk4 signalling pathway. Moreover, high expression of LOC441204 has been correlated with brain tumour progression, and inhibition of LOC441204 expression resulted in the suppression of tumour cell proliferation in two glioma cell lines.^68^ Hypoxia‐inducible factor 1 ∝‐antisense RNA 2 (HIF1A‐AS2) is a subtype‐specific hypoxia‐inducible lncRNA that was shown to be overexpressed in mesenchymal GBM stem‐like cells (GSCs). In vitro studies demonstrated that down‐regulation of HIF1A‐AS2 led to delayed growth of mesenchymal GSC tumours, survival benefits and impaired expression of HMGA1, a target gene of this lncRNA, also was overexpressed in glioma.^69^, ^70^

There are several other lncRNAs that impact glioma or GSC biology, such as XIST and linc‐POU3F3. Both of these lncRNAs are up‐regulated in glioma tissues and GSCs and could be classified as oncogenes in glioma and in many other cancers. Knockdown of XIST reduced GCS proliferation, migration and invasion as well as tumour growth in nude mice. Mechanistic studies revealed that miR‐152 mediates the tumour‐suppressive effects of XIST silencing on GSCs, probably by direct interaction.^71^ Linc‐POU3F3 is one of the few lncRNAs with evolutionarily conserved sequence and genomic location, and its expression is negatively correlated with its neighbouring gene, POU3F3.^72^ As a TF, POU3F3 is involved in the regulation of key genes in the neurogenic differentiation of stem cells (DELTA1 and SOX2) and is required for maintaining the undifferentiated phenotype of neural precursors.^73^

## 6 TUMOUR‐SUPPRESSOR lncRNAS

The cellular mechanism of tumour‐suppressor lncRNAs consists of inhibiting uncontrolled cellular growth, migration and invasion or inducing apoptosis. MEG3 is highly expressed in normal brain tissue and is down‐regulated in gliomas, inhibiting cell proliferation/DNA synthesis by stimulating expression of p53 protein and modulating its binding to target gene promoters.^32^, ^47^ Functional studies revealed that restored expression of MEG3 inhibited GBM cell proliferation and induced apoptosis as well as autophagy.^61^ In MEG3 knockout mice, expression of genes involved in the VEGF angiogenic pathway and vascularization in the brain is affected.^74^ The ADAMTS9‐AS2 antisense transcript of ADAMTS9 gene was shown to be significantly down‐regulated in glioma tissues and was negatively correlated with tumour grade and prognosis. The ADAMTS9 gene is widely expressed in foetal and adult tissues, functioning as a tumour suppressor gene in diverse cancer types.^75^ Overexpression of ADAMTS9‐S2 in glioma cells inhibited cell migration and invasion, while ADAMTS9‐S2 silencing increased the malignancy of GBM cells, validating its tumour suppressor activity.^76^

GSCs can initiate and maintain glioma growth and may confer resistance to conventional therapies. Several studies have revealed that in general, cancer stem cells can participate in the same reprogramming process as that of iPSCs in maintaining their cell population.^77^, ^78^ Interestingly, linc‐ROR, which directly interacts with pluripotency stem cell TFs (SOX2, OCT4 and NANOG) and controls the process of stem cell reprogramming, was identified as an oncogene in several types of cancer, but in glioma, linc‐ROR possesses a tumour‐suppressor function. One study reported that linc‐ROR expression was significantly down‐regulated in glioma tissues compared to that in adjacent tissues and that its silencing significantly enhanced the proliferation of glioma cells and the spheronization of GSCs. At the same time, linc‐ROR negatively modulates the expression of stem cell factor KLF4, and its restored expression in GBM cells induces the inhibition of GSC proliferation and self‐renewal.^79^ A previous study showed that TFs such as SOX2, OCT4, NANOG and KLF4 are co‐overexpressed in GBM.^77^ Therefore, GBM malignancy could be enhanced by the acquisition of pluripotent stem cell (PSC) properties. It is possible that TFs that regulate the self‐renewal and pluripotency of stem cells have similar regulatory functions in gliomas.

LincRNA‐p21 is a p53‐dependent transcriptional target gene involved in normal cellular proliferation, cell cycle and stem cell reprogramming.^30^ In iPSC, lincRNA‐p21 prevented reprogramming by activating epigenetic markers to induce heterochromatin at pluripotency gene promoters.^80^ Moreover, lincRNA‐p21 is down‐regulated in many types of cancer, including gliomas and GSCs, behaving as a tumour suppressor. Functional studies revealed that lincRNA‐p21 displays anti‐EMT activity and suppresses Wnt/β‐catenin signalling, leading to decreased cell viability, self‐renewal and glycolysis in GSCs. The overexpression of lincRNA‐p21 dramatically decreased self‐renewal capacity and tumorigenicity of GSCs in xenograft mice, and its silencing induced β‐catenin overexpression, leading to increased stemness and radioresistance of GSCs.^81^

To date, sufficient evidence has been gathered and tested regarding lncRNA involvement in glioma development. With special attention to particular areas of glioma biology, such as cancer stem cells, lncRNAs are bound to become active players in patient care, such as for prognostic and/or preventative purposes, or even for targeted treatment. Discussion will focus on epigenetic mechanisms influencing lncRNA dysregulation, as well as lncRNA‐driven epigenetic changes in glioma.

## 7 LNCRNA EPIGENETICS IN GLIOMA

Epigenetics refers to the heritable and reversible modifications that affect gene expression and genomic stability without altering the DNA sequence.^82^ The main epigenetic mechanisms comprise chemical modifications of DNA and histones (cytosine methylation, histone posttranslational modifications), chromatin remodelling and nucleosome positioning. These changes are reversible and controlled by enzyme complexes directly connected to metabolic and signalling pathways as well as sensors of extra‐ and intracellular microenvironments.^82^ Ongoing research continues to uncover novel epigenetic pathways, including the important roles of non‐protein‐coding RNA transcripts (microRNAs and lncRNAs) in gene regulation as part of a larger epigenetic network.^83^

Tumorigenesis results from a complex interplay of both genetic alterations and epigenetic changes affecting various cellular processes. In primary GBM, 80% of studied cases exhibit severe global hypomethylation of DNA.^6^ The CpG island methylator phenotype is frequently found in secondary and recurrent GBM tumours and was correlated with high rates of IDH1 and TP53 mutations, early age of diagnosis and better prognosis.^4^, ^6^

In GBM, genome‐wide sequencing has identified an enormous number of mutations in epigenetic regulatory genes, including histone deacetylase (HDAC) 2 and 9, histone demethylases and methyltransferases.^84^ Furthermore, globally altered expression levels of epigenetic modifiers have been linked to prognostic markers in glioma patients. Class II and class IV HDACs display decreased mRNA expression in GBMs compared to those in low‐grade astrocytomas and normal brain samples, and overall histone H3 hyperacetylation has been linked to tumour recurrence and progression in GBM.^85^

## 8 EPIGENETIC CONTROL OF LNCRNA EXPRESSION

Dysregulated lncRNAs are present in every step of tumour initiation and progression, but the molecular mechanism of different patterns of lncRNA expression in cancer remains unknown. Epigenetic changes could explain the stable and rapid alterations of oncogenic and tumour suppressor acting lncRNA expression without requiring any additional genetic mutation. Several studies demonstrated that lncRNA expression level could be controlled by methylation status at the promoter region and by direct interaction with histone modifiers and chromatin‐remodelling complexes.^86^, ^87^, ^88^ Zhi et al performed epigenome‐wide association studies profiling the DNA methylation status of lincRNA genes in thousands of tumour samples and in normal tissue;^89^ 2461 lincRNA genes were classified as prone to methylation (PM) or resistant to methylation (RM) and were further associated with cancer status, subtypes or prognosis. Interestingly, promoters of RM lincRNA genes are evolutionally conserved among species and ubiquitously expressed in normal tissues.^89^

Recently, one study illustrated that the down‐regulation of MEG3 expression in glioma is due to hypermethylation of its promoter. Treatment of glioma cells with the DNA methylation inhibitor 5‐aza‐dC decreased aberrant promoter hypermethylation and prevented loss of MEG3 expression. In addition, DNMT1 expression was inversely correlated with MEG3 transcript, supporting DNMT1's involvement in MEG3 dysregulation in glioma.^61^ Importantly, the same association of MEG3 expression with promoter methylation status has been observed in hepatocellular cancer, and direct involvement of miR‐29a, a DNMT1 and DNMT3B epigenetic modulator, was also demonstrated.^90^ The activity of tumour suppressor ADAMTS9‐AS2 is also controlled by DNA methylation in glioma. Additionally, the expression of its transcript was positively correlated with ADAMTS9 and DNMT3A expressions.^76^ A recent study showed that the lncRNA LOC285758 expression is regulated by DNA methylation and differs within glioma subtypes, with overexpression and higher promoter hypomethylation in GBM.^91^

Histone modifications play an essential role in the regulation of lncRNA expression. LncRNA promoters exhibit specific histone marks, including methylated H3K4, H3K27, H3K36, and acetylated H3K9 and H3K27, suggesting that they undergo epigenetic regulation similar to that of PCG.^21^ Genome‐wide analysis in different cell and tissue types has revealed that highly expressed lncRNAs were enriched in active H3K4me3 and H3K36me3 sites, whereas lowly expressed lncRNAs were highly marked by H3K27me3. Zhao et al proposed an integrative method to identify epigenetically altered lncRNAs and their associated genes in GBM, GSCs and normal controls. By combining RNA and ChIP‐seq data for H3K4me3 and H3K27me3 marks, the authors found that many disease‐related lncRNAs exhibit aberrant epigenetic modification, and their neighbouring genes are enriched for polycomb repressive protein 2 (PRC2)‐binding sites.^92^ As an example, foetal tissues and GBM highly express HOTAIRM1,^32^ showed a gain of H3K4me3 in GSCs compared to that in GBM tissue. Epigenetically activated HOTAIRM1 can also negatively or positively affect the expression of its neighbouring genes. Indeed, previous studies have shown that HOTAIRM1 plays key roles in myeloid differentiation by *cis‐*regulating the expression of neighbouring homeobox transcription factor A (HOXA) genes.^30^ Thus, it is possible for H3K4me3‐activated HOTAIRM1 to contribute to the development of GBM by enhancing the expression of neighbouring HOXA1, an oncogene present in several cancers.

Mechanistic experiments illustrated that H3 hyperacetylation at lysine 9 (H3K9ac) in the promoter region may account for up‐regulation of CRNDE, a key lncRNA involved in neurogenesis and probably also in gliomagenesis. H3K9ac is a known mark for actively transcribed promoters, and the acetyltransferase P300/CBP protein responsible for its acetylation was shown to be recruited to the CRNDE promotor in GBM cells, while being absent in normal glial cells.^51^

Epigenetic modulators, such as BRD4 (member of BET‐bromodomain and extra terminal domain) proteins, with histone acetyltransferase and chromatin remodelling functions up‐regulate HOTAIR expression in GBM cells. Chromatin immunoprecipitation analysis demonstrated that BRD4 binds to the HOTAIR promoter, suggesting that BET proteins can directly regulate HOTAIR expression. Moreover, treatment of GBM cells with the BET inhibitor I‐BET151 restored the expression of several other GBM down‐regulated lncRNAs. Conversely, overexpression of HOTAIR in conjunction with I‐BET151 treatment abrogates the anti‐proliferative activity of the BET bromodomain inhibitor in GBM cells.^93^ The large number of epigenetic dysregulated lncRNAs in glioma reveals that epigenetic inhibitors may induce anti‐tumour effects on brain cancer through modulation of lncRNA networks.

**Figure 3:**
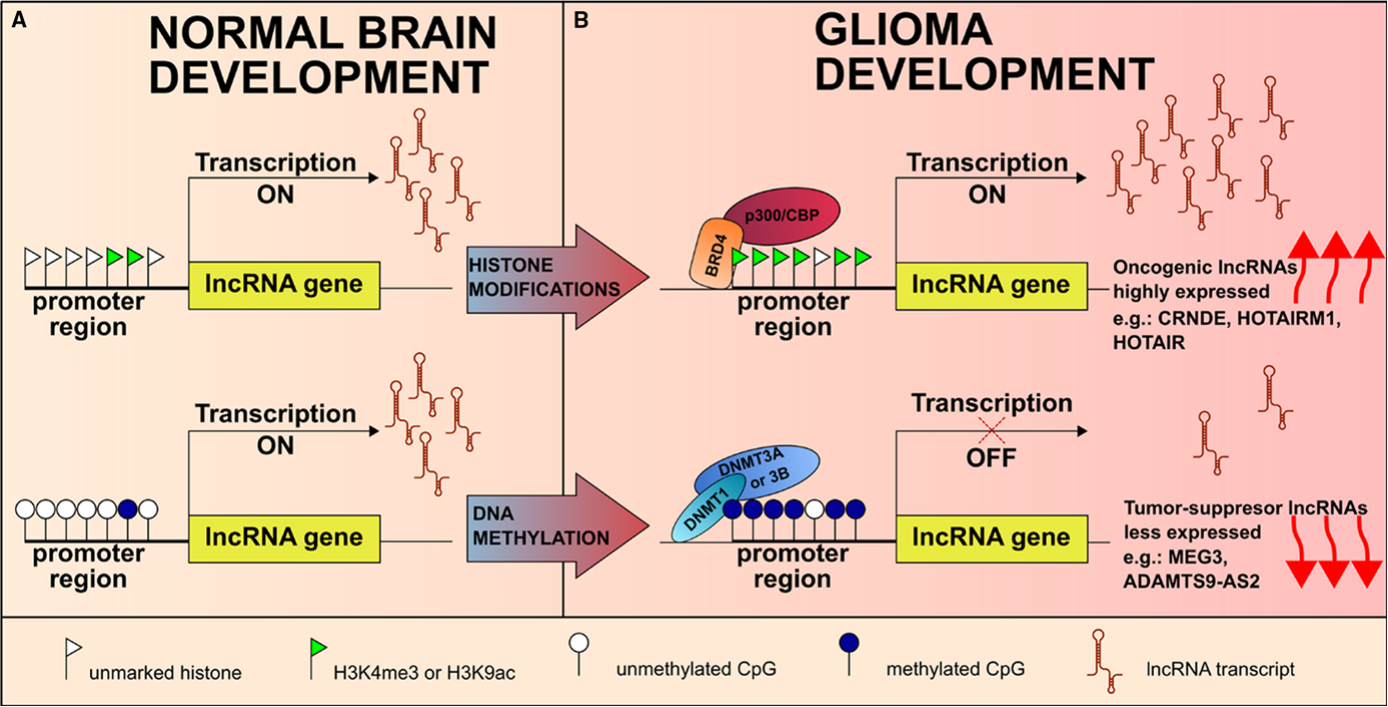
Examples of Epigenetic Modulation of Dysregulated LncRNA Expression in Glioma. A, Epigenetic modulation of lncRNA expression in normal brain development. B, Upper panel: lncRNAs expression are up‐regulated by epigenetic mechanisms: histone modifier p300/CBP protein enhances acetylation of H3K9 at the promoter region of CRNDE gene; gain of H3K4me3 transcriptional activated mark at the promoter region of HOTAIRM1 gene and by direct binding of BRD4 protein—an epigenetic modulator that recognizes acetylated lysine tails of histones and brings the transcriptional machineries to promoter region of HOTAIR gene. Lower panel: epigenetic silencing through methylation of CpG islands at the promoter region of tumour suppressors MEG3 and ADAMTS9‐AS2 in glioma

As Figure 3 shows, reactivation of oncogenic lncRNAs or silencing of tumour suppressor lncRNAs by epigenetic mechanisms might be critical for glioma initiation and progression.

LncRNAs that are involved in both embryogenesis and glioma biology (e.g: CRNDE, HOTAIRM1, ADAMTS9‐AS2), with expression levels controlled by epigenetic mechanisms, could represent a future option for further studies on epigenetic therapy for glioma patients.

## 9 EPIGENETIC ALTERATIONS INDUCED by lncRNAs

As presented above, overexpression or repression of key lncRNAs in gliomas can be mediated by aberrant DNA methylation or histone modification at their gene promoters. Thus, dysregulated expression of such lncRNAs may contribute further to tumorigenesis by inducing aberrant epigenetic modifications to gene regulatory mechanisms.

Emerging evidence indicates that lncRNAs constitute a network of epigenetic modulators by forming platforms for chromatin remodelling complexes and TFs capable of regulating the transcriptional state of the lncRNAs’ controlled genomic locus.^4^, ^10^ *Cis*‐acting lncRNAs adjacent to loci where they are transcribed act by transcriptional interference or by modifying chromatin. Transcriptional interference allows inhibition of preinitialization complexes and interaction with transcription factors. *Trans*‐lncRNAs operate independently of complementarity sequences, acting at larger distance on many genomic loci via specific DNA motifs. Hence, lncRNAs reconfigure differential chromatin states, inducing large‐scale changes in gene expression that can dictate cell malignancy.^82^, ^87^

Almost 20% of identified lincRNAs reportedly bind to PRC1 or PRC2, whose subcatalytic units (EZH2, SUZ12, CoREST) interact by altering histone code and methylation profile, mainly establishing repressive chromatin states marked by H3K27me3.^12^ CRNDE regulates gene expression via interacting with EZH2 and CoREST, a repressor of neuronal genes.^50^ EZH2 is up‐regulated in GBM and appears to be necessary for maintenance of GSC pluripotency and glioma cell proliferation. Thus, as the biology of glioma cells could resemble that of neural precursors, epigenetic modifications likely play a key part in neurogenesis and brain tumorigenesis.

HOTAIR functions as a scaffold for PRC2 and the LSD1/CoREST/REST complex as well as guiding these complexes to their endogenous targets and so activating pro‐oncogenic signalling pathways.^93^ HOTAIR regulates cell cycle progression in glioma cells via interaction with EZH2, and inhibition of HOTAIR represses GBM tumour growth in vivo.^94^ Additionally, PRC2 binding NEAT1 silencing in GBM cells was correlated with decreased H3K27me3 levels in PCGs as part of the Wnt/β‐catenin signalling pathway, such as Axin2.^57^

Another example of complex epigenetic mechanism of gene regulation by lncRNAs is TUG1. In brain development, by binding to PRC2 components, TUG1 selectively regulates neuronal differentiation‐associated genes (NGF, NTF3 and BDNF). In gliomas, TUG1 promotes locus‐specific methylation of histone H3K27 via YY1 binding.^67^ YY1 is a TF capable of selectively binding HDACs or histone‐acetyltransferases in order to activate or repress gene promoters. The YY1 and PRC2 binding regions of TUG1 are evolutionarily conserved between mouse and human, suggesting their importance for TUG1 function in tumorigenesis processes.^25^

Several lncRNAs have been reported to modulate DNA methylation in both normal development and disease. The highly evolutionarily conserved linc‐POU3F3 neighbours the POU3F3 gene that regulates key TFs in neuronal differentiation.^72^ The CpG islands near the POU3F3 gene are highly methylated in glioma and other cancers. Li W et al have demonstrated that linc‐POU3F3 physically interacts with EZH2, bringing DNMTs to the promoter region in oesophageal squamous cell carcinoma.^95^ Thereby, this evolutionarily conserved linc‐RNA epigenetically modulates neighbouring gene expression, which could be its common mechanism of action in cancers.

Understanding how lincRNAs regulate the reprogramming of PSCs could help unveil mechanisms for CSC malignancy. However, two dysregulated lncRNAs in glioma, linc‐RoR and TUNA, have been reported to promote reprogramming efficiency. Linc‐ROR acts as a sponge for miRNAs that target pluripotency regulators, and TUNA directly interacts with RNA‐binding proteins at the promoters of TFs, such as NANOG and SOX2, where transcription is activated by deposition of the active histone mark H3K4me3.^43^ Interestingly, the glioma tumour suppressor lincRNA‐p21 prevents somatic cell reprogramming by sustaining H3K9me3 and/or CpG methylation at pluripotency gene promoters or by direct association with SETDB1, a histone‐lysine N‐methyltransferase protein or DNMT1.^80^

New findings concerning lncRNA involvement in glioma are beginning to be incorporated into clinical trial data. There are several different ways in which lncRNAs may impact clinical research and patient management. Some data correlate the expression of certain lncRNAs with patient prognosis.^96^ Genome‐wide lncRNA expression profiles in glioma patients have revealed that dysregulated lncRNA expression plays important roles in the tumorigenesis and malignant progression of this cancer, and several lncRNAs are related to the prognosis of patients with GBM.^45^ Circulating levels of HOTAIR are significantly correlated with high‐grade brain tumours, and one study demonstrated that this lncRNA could be considered a novel prognostic and diagnostic biomarker for GBM.^97^ Moreover, in vitro and in vivo studies have demonstrated that aberrant expression of lncRNAs is correlated with response to therapy in gliomas. For example, knockdown of MALAT1 increases permeability of the blood‐tumour barrier, which might contribute to the improvement of therapeutic strategies,^98^ while restoration of CASC2 expression up‐regulates PTEN and can increase glioma sensitivity to temozolomide‐based chemotherapy.^99^

By deciphering the molecular mechanisms underlining the mode of action of lncRNAs and by integrating knowledge from other closely related fields, such as epigenetic gene regulation and stem cell biology, many of the unknown aspects of cancer will begin to be understood.

## 10 CONCLUSION AND PERSPECTIVES

The central role played by lncRNAs in numerous critical biological processes may reflect the connections between lncRNAs and the regulation of developmental and physiological processes, whose disruption can lead to cancer. One of the most fundamental roles of epigenetics is the guidance of cellular differentiation during development, and emerging data show the involvement of epigenetic mechanisms in cancer. Multiple changes in gene expression that are directed by epigenetic machinery can explain how malignant cellular processes can arise and hijack normal development signalling pathways.

The very large abundance of lncRNAs in the brain and the link between several lncRNAs involved in normal development and glioma initiation and progression opens a new pathway in investigating lncRNAs potential in diagnosis and treatment of brain tumours.

The genetic and epigenetic heterogeneity of gliomas likely underlies their inherent adaptability and resistance to therapies. Altered epigenetic defects that accumulate in cancer are potentially reversible. Treating tumours with demethylating agents or histone deacetylase inhibitors and down‐regulating onco‐miRNAs expression can activate silenced tumour suppressor genes, all of which may have clinical value. Genetic mutations are almost impossible to reverse; however, epigenetic pathways can be important therapeutic targets. Until now, a major unsolved issue with epigenetic therapy for cancer was target specificity. Through their peculiar features, such as tissue and genomic location specificity, and their modular structure interacting with several proteins involved in epigenetic mechanisms, lncRNAs might yield novel approaches for specific epigenome‐targeted therapies for gliomas or novel biomarkers for diagnostic, prognostic and monitoring purposes.

## CONFLICTS OF INTEREST

The authors confirm that there are no conflicts of interest.
